# The B chromosomes of the African cichlid fish *Haplochromis obliquidens *harbour 18S rRNA gene copies

**DOI:** 10.1186/1471-2156-11-1

**Published:** 2010-01-05

**Authors:** Andréia B Poletto, Irani A Ferreira, Cesar Martins

**Affiliations:** 1 UNESP - Universidade Estadual Paulista, Instituto de Biociências, Departamento de Morfologia, Botucatu, SP, Brazil

## Abstract

**Background:**

Diverse plant and animal species have B chromosomes, also known as accessory, extra or supernumerary chromosomes. Despite being widely distributed among different taxa, the genomic nature and genetic behavior of B chromosomes are still poorly understood.

**Results:**

In this study we describe the occurrence of B chromosomes in the African cichlid fish *Haplochromis obliquidens*. One or two large B chromosome(s) occurring in 39.6% of the analyzed individuals (both male and female) were identified. To better characterize the karyotype and assess the nature of the B chromosomes, fluorescence *in situ *hybridization (FISH) was performed using probes for telomeric DNA repeats, 18S and 5S rRNA genes, SATA centromeric satellites, and bacterial artificial chromosomes (BACs) enriched in repeated DNA sequences. The B chromosomes are enriched in repeated DNAs, especially non-active 18S rRNA gene-like sequences.

**Conclusion:**

Our results suggest that the B chromosome could have originated from rDNA bearing subtelo/acrocentric A chromosomes through formation of an isochromosome, or by accumulation of repeated DNAs and rRNA gene-like sequences in a small proto-B chromosome derived from the A complement.

## Background

Perciformes fishes are the largest order of vertebrates and include the family Cichlidae, which represents the most species-rich vertebrate family, with more than 3,000 species [1]. Cichlid fishes are native to tropical areas of the Americas, Africa and Asia and have undergone spectacular adaptive radiation in the lakes of East Africa (Victoria, Tanganyka, and Malawi) [2]. This group of fishes is an important model for evolutionary processes, mostly because their great phenotypic diversity has evolved in a short period of time, among lineages with similar genomes [3-5]. In addition, some species of Cichlidae, such as the tilapiines *Oreochromis*, *Tilapia *and *Sarotherodon*, are very important for aquaculture [6]. Because of their economic and evolutionary importance, cichlid fishes have been the focus of several genetic and genomic studies [7]. On the other hand, most cytogenetic studies in the group have been directed to *Oreochromis niloticus *and few data are available for other cichlid species. Cytogenetic data have shown that most cichlids from Central and South America have 48 chromosomes, while African species have 44 chromosomes (reviewed in [8]), with some characteristic chromosome pairs within each group.

Many animal and plant species have B chromosomes, also known as accessory, extra or supernumerary chromosomes. B chromosomes are additional and mostly dispensable chromosomes that are present in some individuals in some species. Most of these chromosomes probably arose from A chromosomes, but have followed their own evolutionary pathways [9]. Furthermore, B chromosomes have irregular and non-Mendelian modes of inheritance and are not believed to undergo recombination with any members of the basic A chromosome set [10]. They are mostly heterochromatic [11] and may have originated through chromosomal breakage and fusion of A chromosome(s) in a single species or by hybridization between species [12].

The occurrence of B chromosomes has been described for species of diverse fish groups. In general, the B chromosomes of fishes vary in number from one to eight [13-15]. The size of the B chromosomes can vary greatly, from macro-chromosomes as found in *Astyanax *sp. [16-20] and *Alburnus alburnus *[21,22], medium-sized chromosomes, as in *Rhamdia *sp. [23,24], small-sized chromosomes, as in *Parauchenipterus galeatus *[25], or microchromosomes, as in *Prochilodus *sp. [13,15]. Among cichlids, B chromosomes have been described for only a few species of South American species. They were first described in the male germ cells of *Gymnogeophagus balzanii *[26] and later found in *Geophagus brasiliensis, Cichlasoma paranaensis *and *Crenicichla niederleinii *[27]. B microchromosomes have also been described for *Cichla monoculus*, *Cichla *sp. and *Crenicichla reticulata *[28]. The presence of B chromosomes among the African cichlids has not previously been reported.

Despite being distributed in several fish taxa, the genomic nature and genetic behavior of B chromosomes are still poorly understood. In this study, we performed molecular cytogenetic analysis on the African cichlid fish *H. obliquidens*, in order to better understand the chromosomal organization and meiotic behavior of B chromosomes present in individuals of this species.

## Results

### Basic cytogenetic analysis

**Figure 1:**
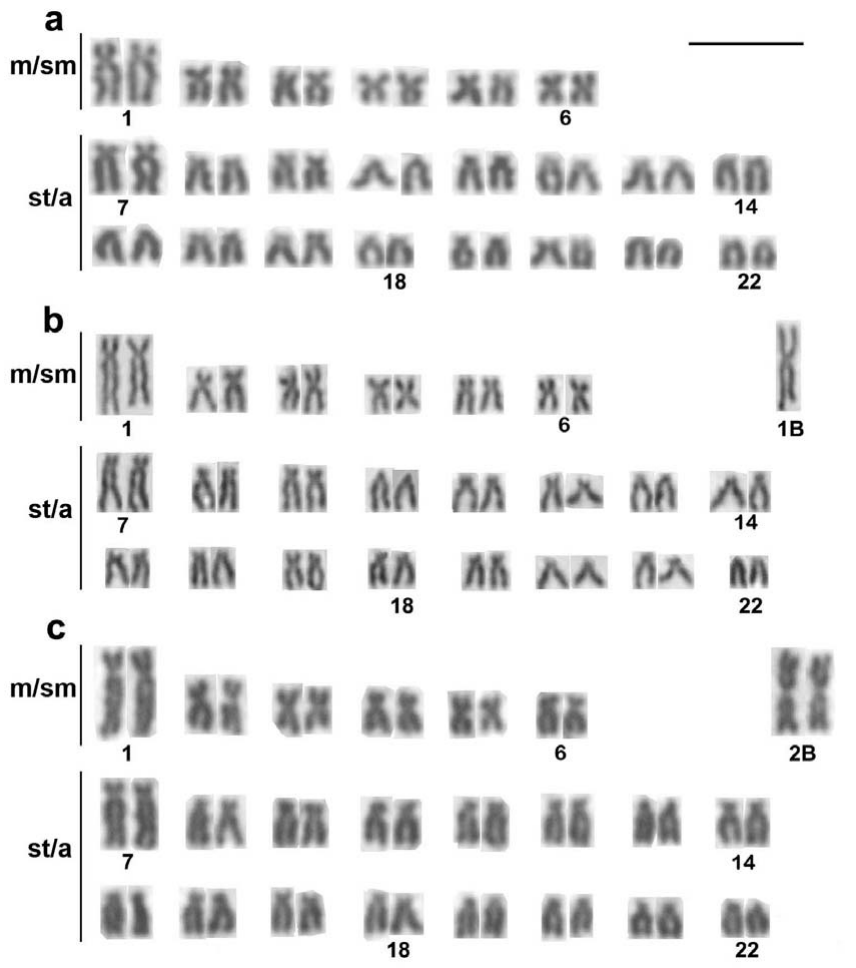
**Karyotypes of *H. obliquidens *without B chromosomes (a), with one extra chromosome (b) and with two extra chromosomes (c)**. The meta-submetacentric (m/sm) and subtelo-acrocentric (st/a), and the B chromosomes (1B and 2B) are indicated. Scale bar indicates 5 μm.

Approximately 30 metaphase spreads were analyzed per specimen to determine the diploid chromosome number and karyotype structure. *H. obliquidens *exhibited diploid chromosome numbers of 2n = 44 (12 m/sm and 32 st/a), without sex-related heteromorphism (Figure 1a). Two outstanding large pairs of chromosomes (pairs 1 and 7, Figure 1) are characteristic. One or two large metacentric B chromosomes (Figure 1b, c) were present in 38 out of 96 analyzed specimens (Table 1). In these specimens the B chromosomes were present in 100% of the analyzed cells.

**Table 1:** B chromosome distribution in the analyzed *H. obliquidens *stock.

Number of B chromosomes	Total sample	Male	Female	Not sexed	% of total sample analyzed
0	58	31	17	10	60.41%
1	30	10	13	07	31.25%
2	08	05	03	-	8.33%

**Figure 2:**
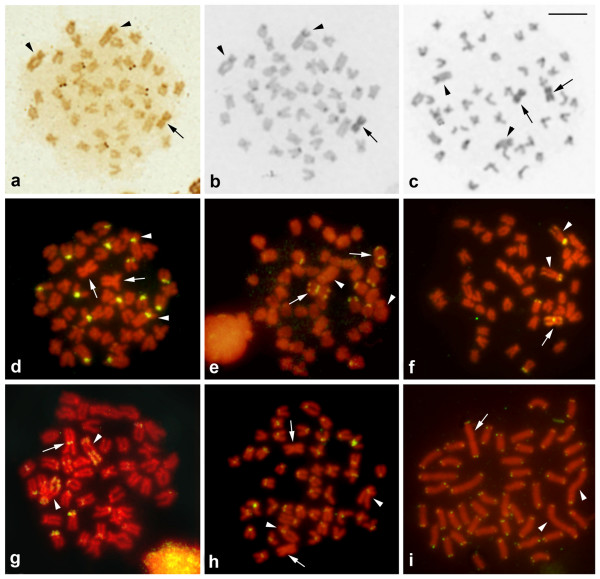
**Metaphasic spreads of *H. obliquidens *after silver staining (a), C-banding (b and c), and *in situ *hybridization with 5S rDNA (d), 18S rDNA (e), BAC-C4E09 (f), BAC-C5E01 (g), SATA satellite DNA (h) and telomeric (GGGTTA)_n _DNA (i)**. (a) and (b) are sequential metaphases. Arrows indicate the B-type chromosomes and arrowheads indicate the chromosome pair 1. Scale bar indicates 5 μm.

Active NORs were identified in the short arms of five chromosomes in the A complement, but were absent in the B chromosomes (Figure 2a). Constitutive heterochromatin was detected in the pericentromeric regions of most A chromosomes, in the short arm of the chromosome pair 1, as well as on the entire length of B chromosomes (Figure 2b, c).

### Cytogenetic mapping of repeated DNAs

The 5S rDNA probe generated *in situ *hybridization signals in the centromeric area of 15 acrocentric chromosomes, including the chromosome pairs 1 and 7, not in the B chromosomes (Figure 2d). The 18S rRNA gene probe identified six sites in the short arms of small st/a chromosomes of the A complement. In addition, 18S rDNA clusters were observed in both telomeric regions and pericentromeric areas of the B chromosomes (Figure 2e).

Chromosomal mapping with the clone BAC-C4E09 from the *O. niloticus *genomic library, which contains LINE-like retrotransposons and satellite DNAs [29], labeled the short arms of two pairs of st/a chromosomes of the A complement (one of them being the largest chromosome pair), and evidenced weak signals along the whole extension and a strong cluster in the pericentromeric region of the B chromosomes (Figure 2f). Chromosomal mapping with the clone BAC-C5E01, which also contains repetitive elements of the *O. niloticus *genome, evidenced strong signals in the short arms of some st/a chromosomes, in almost the whole extent of the largest chromosome of the A complement, and in the centromeric region of the B chromosomes (Figure 2g).

Chromosomal mapping with the SATA-satellite DNA isolated from *O. niloticus *labeled the centromeric region of all A chromosomes, but showed no clearly visible signal in the centromeric region of the B chromosomes (Figure 2h), although a faint signal was detected in the centromeric region of few metaphase B chromosomes (Figure 2h).

Chromosome mapping with the telomeric (GGGTTA)_n _probe labeled all the telomeres, including the telomeres of B chromosomes, and no interstitial signals were observed in either A and B chromosomes (Figure 2i).

### Meiotic analysis

**Figure 3:**
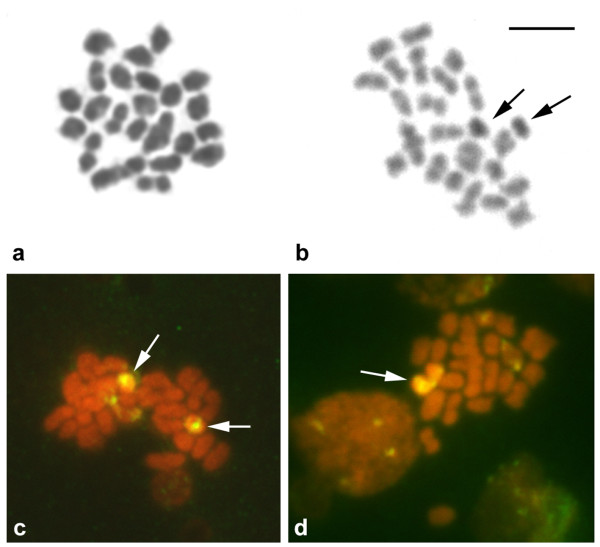
**Meiotic diakinetic spreads from *H. obliquidens *testis cells: Giemsa stained (a), C-banded (b), and probed with 18S rDNA (d)**. Arrows indicate the B-type chromosomes and (d) partially associated B chromosomes. Scale bar indicates 5 μm.

Meiotic chromosomes of mature male *H. obliquidens *with one or two B chromosomes were analyzed. Approximately 10 meiotic plates were analyzed per specimen to determine the haploid chromosome number and meiotic configuration. Chromosomes were Giemsa-stained, C-banded and *in situ *hybridized with the 18S rRNA gene probe (Figure 3). As expected in diakineses, 22 bivalents resembling the 44 A chromosomes and one or two univalent B chromosome(s) were observed (Figure 3a). Although it was difficult to identify the B chromosome(s) among the A elements in the Giemsa-stained meiotic spreads, they were clearly distinguished by C-banding because they appeared totally heterochromatic (Figure 3b). The hybridization of 18S rDNA in diakinetic meiotic cells containing two B chromosomes evidenced the formation of ring-like univalents in most analyzed cells (Figure 3c). However, the two Bs appeared to be slightly associated in few cells (Figure 3d).

## Discussion

### Basic cytogenetic analysis

The standard karyotype we identified is consistent with previous reports on the African cichlids (modal diploid number of 2n = 44) (reviewed in [8]). In addition to the standard cichlid karyotype pattern, a large metacentric B chromosome was observed at high frequency among the sampled animals, representing the first description of B chromosome occurrence in African cichlids. One notable characteristic of the B chromosomes found in this species is its large size, which is almost the same as the largest pair of the A complement. Such a high number of individuals with B(s) (38 out of 96, which accounts for 39.58% of the population), is not commonly observed in studies of B chromosomes, despite the fact that number of individuals with B chromosome can vary in natural fish populations [21]. Considering that the *H. obliquidens *population was obtained from the aquarium trade, the high number of individuals with B chromosomes could be a consequence of founder effects from a small population of breeders. On the other hand, we can also consider that the B chromosomes of *H. obliquidens *could confer some advantage to the carriers and accumulated in the population analyzed. In the same way, it seems plausible to consider that these extra chromosomes occur in natural populations of the species.

### Accumulation of repetitive DNAs in B chromosomes

Heterochromatic B chromosomes as observed in *H. obliquidens *are common among fish species [13,30,31]. However, some cases of euchromatic B chromosomes have also been described in fish, such as *Moenkhausia sanctaefilomenae *[32] and *Characidium *cf. *zebra *[31]. The heterochromatin of the B chromosomes of *H. obliquidens *is enriched in repetitive DNAs, as evidenced by mapping with 18S rDNA, and the clones BAC-C4E09 and BAC-C5E01. These BACs contain repetitive DNAs with similarity to the LINE retrotransposons of the cichlids *Haplotaxodon microlepis *and *O. niloticus*, SATB satellite DNA of *O. niloticus*, and repeated segments of the *Danio rerio *genome [29]. On the other hand, the large number of 5S rDNA sites observed in *H. obliquidens *suggest that most centromeric heterochromatins contain repetitive 5S rRNA gene-like copies that are not clustered in the Bs. Repetitive satellite DNA originated from 5S rDNA was previously described in the fish *Hoplias malabaricus *[33]. The organization and evolution of tandem repetitive DNAs is governed by particular patterns of evolution such as unequal exchange, transposition, RNA-mediated transposition and gene conversion causing the origin of variant copies of rDNA repeats in the genome. These mechanisms could be responsible for the dispersion and accumulation of copies of 5S rDNA repeats in specific areas of the genome (reviewed in [33]). The genomic structure of 5S rDNA repeats should be better investigated in the genome of *H. obliquidens *to clarify the origin and possible role of such dispersed pattern.

The presence of repeated sequences with similarities to retrotransposons in a B chromosome has been previously reported for the fish *Astyanax scabripinis *[34], as well as for the B chromosome of the parasitoid wasp *Nasonia vitripennis *[35,36]. A retrotransposon also appears to be involved in the transposition of chloroplast DNA into the repeated element Bd49 of the B chromosomes of *Brachycome dichromosomatica *[37]. The repeated elements are thought to accumulate in the heterochromatic regions of chromosomes, which are characterized by lower gene density and reduced recombination [38]. The absence of recombination of B chromosomes of *H*. obliquidens, as shown by the univalent conformation that they assume during meiosis, may facilitate the accumulation of repeated DNAs. Repetitive DNAs were long considered to be junk DNA because they had no clearly identified function [39,40]. However, further studies of their accumulation in specific genomic areas, causing chromosome rearrangements through breakages, deletions, inversions and amplifications [41,42], have shown they can operate at the chromosomal level in the speciation process. These recent findings have suggested that junk DNA may be one of the major drivers of genome evolution [43]. Consequently, the study of repetitive DNA in B chromosomes could yield new insights into the evolutionary mechanisms involved with the origin and evolution of particular chromosome elements.

### Presence of 18S rRNA genes copies in B chromosomes

Another remarkable characteristic described here is the presence of 18S rRNA gene copies in both telomeric regions and centromeric areas of B chromosomes. The presence of rDNA sequences in the B chromosome suggests its possible origin from A chromosomal elements that harbor clusters of 18S rRNA genes. It is believed that extra rDNA copies could initially increase transcription rates and can present an advantage to the bearers of the B chromosome (reviewed in [12]). On the other hand, the 18S rDNA sites in the B chromosomes of *H. obliquidens *appeared not stained by silver nitrate, suggesting that they are not actually being transcribed, as silver has a strong affinity for the proteins that are assembled during nucleolus formation [44]. The presence of non-transcribed rRNA genes was described in the B chromosomes of the black rat *Rattus rattus *[45]. In this case, the B chromosomes seem to have arisen from A chromosomes that harbour rRNA genes and was subsequently invaded by repetitive sequences with the dispersion of rRNA genes throughout the B chromosome [45]. On the other hand, active rRNA genes were observed in the telomeric regions of B chromosome of the rodents *Akodon montensis *and *Oryzomys angouya *[46]. In *H. obliquidens *the rRNA genes accumulated in the centromeric and terminal regions of the B chromosomes and the repeated DNAs are dispersed throughout the entire B chromosomes with enrichment in the centromeric area. Considering that no silver signal was detected on the Bs of any cell of *H. obliquidens *we can hypothesize that the rRNA genes in the B chromosome are permanently inactive representing variant copies (pseudogenes) of the true rRNA genes.

Previous studies have found evidence of dispersed or clustered elements similar to 45S rDNA in several eukaryotic genomes. These elements have mainly been characterized as noncoding, small-unit tandem repeats of variable copy number. Such elements have been identified in various eukaryotic species, including yeast [47], animals [48], and plants [49]. Moreover, some authors have hypothesized that ribosomal cistrons could undergo active transposition followed by tandem amplification. This transposition event could be facilitated by recombination between preexisting repetitive DNAs in the constitutive heterochromatins and/or rDNA clusters. Repeated DNAs seem to be associated with ribosomal DNAs in *A. scabripinnis*, facilitating recombination between terminal segments in the same chromosome pair or between terminal portions from non-homologous chromosomes [34,50]. The results presented here for *H. obliquidens *and previously observed for other vertebrates [45,46] show rRNA gene-like copies may have originated from the true rRNA genes and further accumulated in the B chromosomes through the same mechanism that drives the evolution of repeated DNAs. These mechanisms could have been involved in the distribution of rDNA copies in B chromosomes of *H. obliquidens*. Moreover, the regions of chromosomes with repetitive DNAs, including 18S rDNA copies, are among the last regions of the genome to segregate during cell division, as previously reported in yeast [51,52], and may therefore be involved in the non-disjunction and accumulation of the B chromosome in *H. obliquidens *cells.

### Possible origin and evolution of the B chromosomes

The meiotic behavior of the B chromosomes show they do not associate with any of the A chromosomes and do not form true bivalents in the cell when two Bs are present during prophase I of meiosis. The two Bs appeared slightly associated in metaphasic images of a few cells, probably as consequence of the similarity in their repetitive DNA content. The chromosomes appeared to associate themselves through the rDNA sequences in their terminal regions. Indeed, this slight association was rarely seen in the majority of analyzed meiosis plates, and it could be favored due to the accumulation of the 18S rDNA repeats on both B chromosome arms. Furthermore, Bs form a ring univalent structure, that appears when their arms are engaged in a chiasmatic union, and this type of meiotic chromosome conformation has been previously interpreted as an indication of an isochromosome origin. Among fish species, the large B chromosomes of *Astyanax scabripinnis *[34] and *Alburnus alburnus *[21] represent examples of isochromosome origin. Considering that SATA centromeric satellite could be involved with centromeric function in the cichlid chromosomes [53,54], the absence of clear visible SATA satellite cluster in the Bs of *H. obliquidens *suggests that this extra element does not regularly segregate during the cell division process as previously reported for the plant *Lilium callosum *[55] and the animal *Myrmeleotettix maculatus *[56,57], and other examples from grasshoppers [58].

Moreover, the presence of conserved repetitive sequences between the A and B chromosomes suggests that the B element could have originated from the A genome. In the same way, the presence of 18S rRNA gene-like sequences in this accessory element indicates that its possible source might be related to st/a chromosomes of A complement that bear ribosomal genes. The st/a chromosomes bearing 18S rRNA sites also harbours repetitive DNAs that are shared with the Bs. It could have originated as an isochromosome by misdivision of a centromere followed by chromatid nondisjuction. Further chromosomal rearrangements and accumulation of repetitive DNAs could have resulted in the observed B chromosome. The symmetric distribution of 18S rDNA and repetitive sequences between the B arms also support an isochromosome origin. Alternatively, Robertsonian fusion between two acrocentric chromosomes bearing rDNA sites could also account for the origin of the B chromosomes. The absence of telomeric interstitial signals in the B chromosome indeed corroborates the first hypothesis, demonstrating that the B chromosome was not caused by a simple translocation. Another hypothesis is that the B element could have originated through the amplification and accumulation of repeated DNAs from a proto-B-chromosome that was generated from a small chromosome fragment of the A complement generated after non-disjunction during meiosis.

## Conclusion

The presence of repeated DNAs (including rRNA gene copies) in specific chromosomes of the A complement suggests that Bs could have arisen from genomic segments of such A elements. This proto-B chromosome(s) would then be freed from the selection pressures that act on the maintenance of standard chromosomes and could have increased of size due to the accumulation of repetitive DNAs in the absence of recombination.

## Methods

### Specimens, and chromosome preparation and banding

We analyzed ninety-six specimens of *H. obliquidens *(46 males, 33 females and 17 of undetermined sex) obtained from the aquarium trade in Botucatu, SP, Brazil (Table 1). The animal experiments were performed with the approval of the appropriate ethics committee of UNESP - São Paulo State University. Metaphase chromosomes were obtained from cells of the anterior kidney following *in vivo *treatment with colchicine at 0.025% (1 ml/100 g of body weight) according to the air-drying method [59]. Heterochromatin was identified by C-banding [60], and the nucleolus organizer regions (NORs) were visualized by silver nitrate staining [61]. Chromosomes were classified as meta/submetacentric (m/sm) and subtelo/acrocentric (st/a), and were organized by decreasing order of size in the karyotype. Meiotic cells from testes were obtained as described by Kligerman and Bloom [62].

### Fluorescence in situ hybridization (FISH)

Fluorescence *in situ *hybridization (FISH) was performed to map repeated DNAs on the mitotic and meiotic chromosomes of *H. obliquidens*. Five DNA probes containing sequences of different classes of repeated DNA were used for chromosome hybridization. (i) 5S rDNA probe: complete repeat units of 5S rDNA of *H. obliquidens *were obtained by the polymerase chain reaction (PCR) with the primers 5SA (5'-TAC GCC CGA TCT CGT CCG ATC - 3') and 5SB (5' - CAG GCT GGT ATG GCC GTA AGC-3') designed from the rainbow trout 5S rRNA sequence [63] and successfully applied for the amplification of 5S rDNA of other cichlids [64,65]. (ii) 18S rDNA probe: a segment of 1,400 base pairs (bp) of the 18S rRNA gene of *H. obliquidens *was obtained by PCR with the primers 18Sf 5'CCG CTT TGG TGA CTC TTG AT and18Sr 5'CCG AGG ACC TCA CTA AAC CA. The 18S primers were designed from the catfish *Ictalurus punctatus *(GenBank accession number AF021880) and have been successfully used to amplify 18S rRNA genes of different fish species [65,66]. (iii) SATA satellite: repeated satellite DNA isolated and cloned from *O. niloticus *[29]; (iv) Telomeric DNA sequences: *in vitro *synthesized oligomers of telomeric repeats (GGGTTA)_7_/(TAACCC)_7_; (v) Clones BAC-C4E09 and BAC-C5E01: Bacterial artificial chromosomes containing several classes of repeated elements from the *O. niloticus *genome [29].

Probes were labeled by nick translation with biotin 14-dATP (Bionick labeling system-Invitrogen). After denaturation of chromosomal DNA in 70% formamide/2× SSC for 40 seconds at 70°C, hybridization mixtures containing 100 ng of denatured probe, 10 mg/ml dextran sulfate, 2× SSC and 50% formamide, in a final volume of 30 μl, were dropped on the slides and the hybridization was performed overnight at 37°C in a 2× SSC moist chamber. Post-hybridization washes were carried out at 45°C in 2× SSC/50% formamide for 15 min, followed by a second wash in 2× SSC for 15 min, and a final wash at room temperature in 4× SSC for 15 min. Detection of hybridized probes was carried out with 0.07% avidin FITC conjugate (Sigma) in C buffer (0.1 M NaHCO_3 _/0.15 M NaCl) for 1 h, followed by two rounds of signal amplification using 2.5% anti-avidin biotin conjugate (Sigma) in blocking buffer (1.26% NaHCO_3_, 0.018% sodium citrate, 0.0386% Triton X-100 an 1% non-fat dried milk) for 30 min. Each treatment with anti-avidin biotin conjugate was followed by a treatment with avidin-FITC. The treatments with avidin-FITC and anti-avidin-biotin were conducted in a 2× SSC moist chamber at 37°C. After each amplification step, the slides were washed three times for 5 min each in blocking buffer at 42°C. Chromosomes were counterstained with propidium iodide diluted in antifade (Vectashield Mounting Medium, Vector). Hybridized chromosomes were visualized using an Olympus BX 61 microscope, and images were captured with a digital camera Olympus DP71 with the software Image-Pro MC 6.0. Karyotypes and metaphases were arranged with Adobe Photoshop 7.0 software.

## Authors' contributions

ABP carried out the cytogenetic analyses and drafted the manuscript. IAF helped in cytogenetic analysis and drafted the manuscript. CM designed and coordinated the study, and drafted and revised the manuscript. All authors read and approved the final manuscript.

## References

[B1] Kocher TD Adaptive evolution and explosive speciation: the cichlid fish model Nat Rev Genet 2004 5 288 298 10.1038/nrg1316 15131652

[B2] Fryer G Iles TD The Cichlid fishes of the great lakes of Africa: Their biology and evolution 1972 Neptune City: TFH Publications

[B3] Won Y-J Sivasundar A Wang Y Hey J On the origin of Lake Malawi cichlid species Proc Natl Acad Sci USA 2005 102 6581 6586 10.1073/pnas.0502127102 15851665 PMC1131877

[B4] Won Y-J Wang Y Sivasundar A Raincrow J Hey J Nuclear gene variation and molecular dating of the cichlid species flock of Lake Malawi Mol Biol Evol 2006 23 828 838 10.1093/molbev/msj101 16461358

[B5] Hulsey CD Function of a key morphological innovation: fusion of the cichlid pharyngeal jaw Proc Biol Sci 2006 273 669 675 10.1098/rspb.2005.3375 16608685 PMC1560081

[B6] Trewavas E Tilapiine fishes of the genera Sarotherodon, Oreochromis and Danakilia 1983 London: British Museum Natural History

[B7] Cnaani A Hulata G Kocher TD, Kole C Tilapias Genome Mapping and Genomics in Fishes and Aquatic Animals 2008 Berlin: Springer-Verlag 101 116 full_text

[B8] Feldberg E Porto JIR Bertollo LAC Val AL, Kapoor BG Chromosomal changes and adaptation of cichlid fishes during evolution Fish adaptations 2003 New Dehli and New York: Science Publishers 285 308

[B9] Beukeboom LW Bewildering Bs: An impression of the 1^st ^B-Chromosome Conference Heredity 1994 73 328 336 10.1038/hdy.1994.140

[B10] Jones RN B chromosomes in plants New Phytol 1995 131 411 434 10.1111/j.1469-8137.1995.tb03079.x 33863119

[B11] Jones RN Rees H B Chromosomes 1982 London: Academic Press

[B12] Camacho JPM Sharbel TF Beukebomm LW B-chromosome Evolution Phil Trans R Soc Lond B 2000 355 163 178 10.1098/rstb.2000.0556 PMC1692730 10724453

[B13] Pauls E Bertollo LAC Evidence for a system of supernumerary chromosome in *Prochilodus scrofa *(Pisces, Prochilodontidae) Caryologia 1983 36 307 314

[B14] Oliveira C Saboya SMR Foresti F Senhorini JA Bernardino G Increased B chromosome frequency and absence of drive in the fish *Prochilodus lineatus* Heredity 1997 79 473 476 10.1038/hdy.1997.186

[B15] Cavallaro ZI Bertollo LAC Perfectti F Camacho JPM Frequency increase and mitotic stabilization of a B chromosome in fish *Prochilodus lineatus* Chromosome Res 2000 8 627 634 10.1023/A:1009242209375 11117359

[B16] Maistro EL Foresti F Oliveira C Almeida-Toledo LF Occurrence of macro B chromosome in *Astyanax scabripinnis paranae *(Pisces, Characiformes, Characidae) Genetica 1992 87 101 106 10.1007/BF00120999

[B17] Salvador LB Moreira-Filho O B chromosome in *Astyanax scabripinnis *(Pisces, Characidae) Heredity 1992 69 50 56 10.1038/hdy.1992.93

[B18] Vicente VE Moreira-Filho O Camacho JPM Sex-ratio distortion associated with the presence of a B chromosome in *Astyanax scabripinnis *(Teleostei, Characidae) Cytogenetic Cell Genet 1996 74 70 75 10.1159/000134385 8893805

[B19] Moreira-Filho O Fenocchio AS Pastori MC Bertollo LAC Occurrence of a metacentric macrochromosome B in different species of the genus *Astyanax *(Pisces, Characidae, Tetragonopterinae) Cytologia 2001 66 59 64

[B20] Ferro DAM Moreira-Filho O Bertollo LAC B chromosome polymorphism in the fish, *Astyanax scabripinnis* Genetica 2003 119 147 153 10.1023/A:1026086501252 14620954

[B21] Ziegler CG Lamatsch DK Steinlein C Engel W Schartl M Schmid M The giant B chromosome of the cyprinid fish *Alburnus alburnus *harbours a retrotransposon-derived repetitive DNA sequence Chromosome Res 2003 11 23 35 10.1023/A:1022053931308 12675303

[B22] Schmid M Ziegler CG Steinlein C Nanda I Schartl M Cytogenetics of the bleak (*Alburnus alburnus*), with special emphasis on the B chromosomes Chromosome Res 2006 14 231 242 10.1007/s10577-006-1038-5 16628494

[B23] Fenochio AS Bertollo LAC Supranumerary chromosome in a *Rhamdia hilarii *population (Pisces, Pimelodidae) Genetica 1990 81 193 198 10.1007/BF00360864

[B24] Fenocchio AS Bertollo LAC Takahashi CS Camacho JPM B chromosomes in two fish species, genus *Rhamdia *(Siluriformes, Pimelodidae) Folia Biol 2000 48 105 109 11291534

[B25] Lui RL Blanco DR Margarido VP Moreira Filho O First description of B chromosomes in the family Auchenipteridae, *Parauchenipterus galeatus *(Siluriformes) of the São Francisco River basin (MG, Brazil) Micron 2009 40 552 559 10.1016/j.micron.2009.03.004 19394233

[B26] Feldberg E Bertollo LAC Discordance in chromosome number among somatic and gonadal tissue cells of *Gymnogeophagus balzanii *(Pisces, Cichlidae) Rev Brasil Genet 1987 7 639 645

[B27] Martins-Santos IC Portela-Castro ALB Julio HF Jr Chromosome analysis of 5 species of the Cichlidae family (Pisces, Perciformes) from the Paraná River Cytologia 1995 60 223 231

[B28] Feldberg E Porto JIR Alves-Brinn MN Mendonça MNC Benzaquem DC B chromosomes in Amazonian cichlid species Cytogenet Genome Res 2004 106 195 198 10.1159/000079287 15292591

[B29] Ferreira IA Martins C Physical chromosome mapping with repetitive DNA sequences in Nile tilapia *Oreochromis niloticus*: evidences for a differential distribution of repetitive elements in the sex chromosomes Micron 2008 39 411 418 10.1016/j.micron.2007.02.010 17395473

[B30] Almeida-Toledo L Foresti F Trajano E Almeida-Toledo S Cytogenetic analysis of the Brazilian blind catfish *Pimelodella kronei *and of its presumed ancestor *Pimelodella transitoria* Caryologia 1992 45 255 262

[B31] Venere PC Miyazawa C Galetti PM New cases of supernumerary chromosomes in characiform fishes Genet Mol Biol 1999 22 345 349 10.1590/S1415-47571999000300010

[B32] Foresti F Almeida-Toledo LF Toledo-Filho SA Supranumerary chromosomes systems, C-banding pattern characterization and multiple nucleolus organizer regions in *Moenkhausia sactaefilomenae *(Pisces, Characidae) Genetica 1989 79 107 114 10.1007/BF00057927

[B33] Martins C Ferreira IA Oliveira C Foresti F Galetti PM Jr A tandemly repetitive centromeric DNA sequence of the fish *Hoplias malabaricus *(Characiformes: Erythrinidae) is derived from 5S rDNA Genetica 2006 127 133 141 10.1007/s10709-005-2674-y 16850219

[B34] Mestriner CA Galetti PM Valentini SR Ruiz IRG Abel LDS Moreira-Filho O Camacho JPM Structural and functional evidence that a B chromosome in the characid fish *Astyanax scabripinnis *is an isochromosome Heredity 2000 85 1 9 10.1046/j.1365-2540.2000.00702.x 10971685

[B35] Mcallister BF Isolation and characterization of a retroelement from a B chromosome (PSR) in the parasitic wasp *Nasonia vitripennis* Insect Mol Biol 1995 4 253 262 10.1111/j.1365-2583.1995.tb00031.x 8825763

[B36] Mcallister BF Werren JH Hybrid origin of a B chromosome (PSR) in the parasitic wasp *Nasonia vitripennis* Chromosoma 1997 106 243 253 10.1007/s004120050245 9254726

[B37] Franks TK Houben A Leach CR Timmis JN The molecular organization of a B chromosome tandem repeat sequence from *Brachycome dichromosomatica* Chromosoma 1996 105 223 230 10.1007/BF02528770 8854881

[B38] Szauter P An analysis of regional constraints on exchange in *Drosophila melanogaster *using recombination-defective meiotic mutants Genetics 1984 106 45 71 6420228 10.1093/genetics/106.1.45 PMC1202246

[B39] Doolittle WF Sapienza C Selfish genes, the phenotype paradigm and genome evolution Nature 1980 284 601 603 10.1038/284601a0 6245369

[B40] Orgel LE Crick FHC Selfish DNA: The ultimate parasite Nature 1980 284 604 607 10.1038/284604a0 7366731

[B41] Lim JK Simmons MJ Gross chromosome rearrangements mediated by transposable elements in *Drosophila melanogaster* Bioessays 1994 16 269 275 10.1002/bies.950160410 8031304

[B42] Dimitri F Arca B Berghella L Mei E High genetic instability of heterochromatin alter transposition of the LINElike 1 factor in *Drosophila melanogaster* Proc Natl Acad Sci USA 1997 94 8052 8057 10.1073/pnas.94.15.8052 9223313 PMC21555

[B43] Biémont C Vieira C Junk DNA as an evolutionary force Nature 2006 443 521 524 10.1038/443521a 17024082

[B44] Sumner AT Chromosome banding 1990 London: Unwin Hyman

[B45] Stitou S Díaz de la Guardia Jiménez R Burgos M Inactive ribosomal cistrons are spread throughout the B chromosomes of *Rattus rattus *(Rodentia, Muridae). Implications for their origin and evolution Chromosome Res 2000 8 305 311 10.1023/A:1009227427575 10919721

[B46] Silva MJJ Yonenaga-Yassuda Y B chromosomes in Brazilian rodents Cytogenet Genome Res 2004 106 257 263 10.1159/000079296 15292600

[B47] Childs G Maxson R Cohn RH Kedes L Orphons: dispersed genetic elements from tandem repetitive genes of eukaryotes Cell 1981 23 651 663 10.1016/0092-8674(81)90428-1 6784929

[B48] Lohe AR Roberts PA An unusual *Y *chromosome of *Drosophila simulans *carrying amplified rDNA spacer without rRNA genes Genetics 1990 125 399 406 2379820 10.1093/genetics/125.2.399 PMC1204028

[B49] Falquet J Creusot R Dron M Molecular analysis of DNA homologous to IGS subrepeats Plant Physiol Biochem 1997 35 611 622

[B50] Mantovani M Abel LDS Mestriner CA Moreira-Filho O Evidence of the differentiated structural arrangement of constitutive heterochromatin between two populations of *Astyanax scabripinnis *(Pisces, Characidae) Genet Mol Biol 2004 27 536 542 10.1590/S1415-47572004000400012

[B51] D'Amours D Stegmeier F Amon A Cdc14 and condensin control the dissolution of cohesin-independent chromosome linkages at repeated DNA Cell 2004 117 455 469 10.1016/S0092-8674(04)00413-1 15137939

[B52] Sullivan M Higuchi T Katis VL Uhlmann F Cdc14 phosphatase induces rDNA condensation and resolves cohesion-independent cohesion during budding yeast anaphase Cell 2004 117 471 482 10.1016/S0092-8674(04)00415-5 15137940

[B53] Franck JPC Wright JM Conservation of a satellite DNA sequence (SATB) in the tilapiine and haplochromine genome (Pisces: Cichlidae) Genome 1992 36 187 194 10.1139/g93-025 8458567

[B54] Franck JPC Kornfield I Wright JM The utility of SATA satellite DNA sequences for inferring phylogenetic relationships among the three major genera of tilapiine cichlid fishes Mol Phyl Evol 1994 3 10 16 10.1006/mpev.1994.1002 7545936

[B55] Kayano H Cytogenetic studies in *Lillium callosum *III. Preferential segregation of a supernumerary chromosome Proc Jap Acad 1957 33 553 558

[B56] Hewitt GM Evolution and maintenance of B-chromosomes Chromosomes Today 1973 4 351 369

[B57] Hewitt GM Meiotic drive for B chromosome in primary oocytes of *Myrmeleotettix maculatus *(Orthoptera-Acrididae) Chromosoma 1976 56 381 391 10.1007/BF00292957 949923

[B58] Camacho JPM Gregory TR B Chromosomes The Evolution of the Genome 2005 San Diego: Elsevier 223 286 full_text

[B59] Bertollo LAC Takahashi CS Moreira-Filho O Cytotaxonomic considerations on *Hoplias lacerdae *(Pisces, Erythrinidae) Brazil J Genet 1978 1 103 120

[B60] Sumner AT A simple technique for demonstrating centromeric heterochromatin Expl Cell Res 1972 75 304 306 10.1016/0014-4827(72)90558-7 4117921

[B61] Howell WM Black DA Controlled silver staining of nucleolus organizer regions with a protective colloidal developer: a one-step method Experientia 1980 36 1014 1015 10.1007/BF01953855 6160049

[B62] Kligerman AD Bloom SE Rapid chromosome preparation from solid tissues of fishes J Fish Res Board Can 1977 43 266 269

[B63] Komiya H Takemura S Nucleotide sequence of 5S ribosomal RNA from rainbow trout (*Salmo gairdnerii*) liver J Biochem 1979 86 1067 1080 115850 10.1093/oxfordjournals.jbchem.a132601

[B64] Alves-Costa FA Wasko AP Oliveira C Foresti F Martins C Genomic organization and evolution of the 5S ribosomal DNA in Tilapiini fishes Genetica 2006 127 243 252 10.1007/s10709-005-4013-8 16850228

[B65] Teixeira WG Ferreira IA Cabral-de-Mello DC Mazzuchelli J Valente GT Pinhal D Poletto AB Venere PC Martins C Organization of repeated DNA elements in the genome of the cichlid fish *Cichla kelberi *and its contributions to the knowledge of fish genomes Cytogenet Genome Res 2009 125 224 234 10.1159/000230006 19738382

[B66] Cioffi MB Martins C Centofante L Jacobina U Bertollo LAC Chromosomal variability among allopatric populations of Erythrinidae fish *Hoplias malabaricus *: mapping of three classes of repetitive DNAs Cytogenet Genome Res 2009 125 132 141 10.1159/000227838 19729917

