# The B chromosome of the cichlid fish *Haplochromis obliquidens *harbors 18S rRNA genes

**DOI:** 10.1186/1471-2156-13-3

**Published:** 2012-01-19

**Authors:** Andréia B Poletto, Irani A Ferreira, Cesar Martins

**Affiliations:** 1 UNESP - Universidade Estadual Paulista, Instituto de Biociências, Departamento de Morfologia, Botucatu, SP, Brazil

## Correction

After the publication of our work [1], we detected that the species focus of the study, *Astatotilapia latifasciata *(Figure 1), was erroneously identified as *Haplochromis obliquidens*. This species was described as *Haplochromis latifasciatus *[2] and later ascribed to the genus *Astatotilapia *[3]. Our mistake comes from the fact that this species is also frequently listed as *Haplochromis "zebra obliquidens" *in the aquarium trade. *Astatotilapia latifasciata *has been reported to occur in Lake Nawampasa a small satellite lake of the much larger Lake Kyoga, and in Lake Kyoga located north of Lake Victoria in Uganda [3].

**Figure 1:**
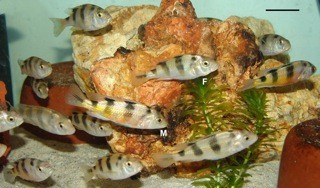
**Specimens of *Astatotilapia latifasciata***. M, male; F, female. Scale bar, 3 cm.
